# Everyday life experiences of family members of individuals with restless legs syndrome: a qualitative interview study

**DOI:** 10.1177/17449871261421131

**Published:** 2026-04-01

**Authors:** Elzana Odzakovic, Maria Björk, Malin Jakobsson, Sandra Öberg, Mattias Georgsson, Lise-Lotte Jonasson, Susanne Knutsson, Martin Ulander, Bengt Fridlund, Anders Broström

**Affiliations:** RN, PhD, Assistant Professor, School of Health and Welfare, Jönköping University, Sweden; RN, RSCN, PhD, Associate Professor, School of Health and Welfare, Jönköping University, Sweden; RN, PhD, Assistant Professor, School of Health and Welfare, Jönköping University, Sweden; RN, PhD, Assistant Professor, School of Health and Welfare, Jönköping University, Sweden; RN, PhD, Assistant Professor, School of Health and Welfare, Jönköping University, Sweden; RN, PhD, Associate Professor, School of Health and Welfare, Jönköping University, Sweden; RN, PhD, Associate Professor, Department of Health and Caring Sciences, Faculty of Health and Life Sciences, Linnaeus University, Sweden; MD, PhD, Assistant Professor, Department of Clinical Neurophysiology, Linköping University Hospital, Sweden; Department of Biomedical and Clinical Sciences, Division of Neurobiology. Linköping University, Sweden; RN, PhD, Senior Professor, Centre for Interprofessional Collaboration within Emergency Care (CICE), Linnaeus University, Sweden; RN, PhD, Professor, School of Health and Welfare, Jönköping University, Sweden; Department of Clinical Neurophysiology, Linköping University Hospital, Sweden; Department of Health and Caring Sciences, Western Norway University of Applied Sciences, Norway

**Keywords:** family members, family nursing, qualitative content analysis, sleep, tiredness, Willis Ekbom Disease

## Abstract

**Background:**

Restless legs syndrome (RLS) is a long-term sensory-motor illness impacting individuals and their family members, yet little is known about the family members’ everyday experiences.

**Aim:**

To explore and describe the everyday life experiences of family members of individuals with RLS.

**Methods:**

An inductive, qualitative exploratory design was used. Telephone interviews were conducted with 25 family members (e.g. partner, child) and analysed using qualitative content analysis.

**Results:**

Family members described adjusting their routines and social activities to accommodate the individual with RLS, often balancing these changes with their own needs. This adaptation affected their everyday lives, as conflicting desires required balancing personal needs with sleep disruptions, resulting in tiredness. Despite these challenges, they employed strategies such as shared activities and self-care to manage the impact on the individuals with RLS.

**Conclusions and contribution to nursing:**

This study highlights the importance of family involvement in RLS care and the need for a family nursing approach in guidelines. Healthcare professionals could invite both family members and individuals with RLS in shared decision-making. Policies and practices could provide resources to support flexible coping strategies and reduce isolation. Future research could explore social support to better understand family members’ experiences.

## Introduction

Family members often experience significant challenges and changes in their life situations when living with an individual who has a long-term illness (Marin-Maicas et al., 2021). Consequently, they must adapt in different ways (Ambrosio et al., 2021). However, adaptation to such situations is a complex process, as individuals navigate their responses to these life changes (Qama et al., 2022). Being diagnosed with a long-term illness like restless legs syndrome (RLS), can be characterised by fluctuations and worsening symptoms (Trenkwalder et al., 2018). The condition can significantly affect a person’s everyday life through mood disturbances and fatigue (Harrison et al., 2021; Odzakovic et al., 2024a; Odzakovic et al., 2025b), which may also impact close relationships with family members (Harrison et al., 2021). The life circumstances of family members of individuals with RLS have not yet been studied.

RLS is a sensory-motor condition (Manconi et al., 2021), with a global prevalence of 3% that increases with age (Broström et al., 2023). The primary complaint is an irresistible urge to move the legs, often accompanied by an unpleasant sensation (Allen et al., 2014). Symptoms are typically more pronounced in the evening and often disrupt sleep (Geng et al., 2022; Odzakovic et al., 2025b). The condition can lead to various challenges for the individual, including sleep disturbances (Xu et al., 2022), excessive daytime sleepiness, depressive symptoms (Chenini et al., 2022), cognitive deficits (Rist et al., 2015), and a reduced quality of life (Broström et al., 2024). Treatment options vary depending on the severity of symptoms; less severe symptoms can be treated solely with the use of non-pharmacological approaches (e.g. massage, physical activity), whereas those with more severe symptoms need pharmacological interventions (e.g. alpha-2-delta ligands, dopaminergic agents and opioids; Gossard et al., 2021; Guay et al., 2020). Unfortunately, pharmacological treatments for RLS reduce symptoms, but do not provide a cure (Winkelmann al., 2025). Moreover, as symptoms can change and increase over time (Allen et al., 2014), it can be difficult to find the right pharmacological treatment and optimise it (Winkelmann et al., 2025), which is why the burden on the individual and family members can gradually increase (Ondo, 2018). Although there is limited research on the effectiveness of non-pharmacological treatment methods in RLS care (Harrison et al., 2019), the use of physical and mental self-care activities (Odzakovic et al., 2024b; Odzakovic et al., 2025a), including support from family members, is likely to be beneficial, as it is in other long-term conditions (Jaarsma et al., 2020; Riegel et al., 2012).

According to the Family Systems Nursing (FSN) theory, having a long-term illness in one family member can influence the entire family unit, affecting the health and well-being of everyone involved (Bell, 2009; Wright and Bell, 2009). Family members actively adapt to these challenges related to long-term illness, providing practical support such as helping with daily activities and coordinating care, as well as emotional support, including belongingness and support (Bell, 2009). Studies on neurological conditions (e.g. Parkinson’s disease, multiple sclerosis) have shown that families must navigate these changes (Pendoni et al., 2024; Ponzio et al., 2024), making decisions based on their experiences of living with a family member with a long-term illness (Årestedt et al., 2014). However, an understanding of the impact of an individual’s RLS on the everyday life of their family members is vital but lacking in relation to RLS. In this study, family members are defined as a self-identified group of two or more individuals, including the individual diagnosed with RLS, who, regardless of their biological or legal relationship (e.g. wife/husband, children), share a sense of family belonging (Wright and Leahey, 2009). Within this definition, the persons with RLS are referred to as the ‘individuals’. This study focuses on the unique challenges faced by family members of individuals with RLS, a condition that often leads to severe symptoms impacting not only the individual affected but also those around them (Allen et al., 2014; Say et al., 2021; Varela et al., 2013). Although research has explored the challenges of being a family member of an individual with a chronic condition for various other chronic conditions (e.g. Chronic Obstructive Pulmonary Disease (COPD), Parkinson’s disease, multiple sclerosis; Fekonja et al., 2024; Pendoni et al., 2024; Ponzio et al., 2024), there is limited understanding of how family members of individuals with RLS experience their everyday life. In a recent study, individuals with RLS reported trying to minimise the impact of their condition on their family members. Their periodic limb movements during sleep often disrupted sleep and caused daytime fatigue, negatively affecting relationships with family members; however, they tried to minimise the impact of their condition on their partners (Harrison et al., 2021). Previous research on family members in an RLS context has primarily examined sleep disturbances (Ondo, 2018), caregiving burden (Say et al., 2021), and psychosocial strain (Varela et al., 2013). Although research exists, little attention has been paid to family members’ daily experiences with RLS. To fill this gap, this study aimed to explore and describe the everyday life experiences of family members of individuals with RLS.

## Methodology

### Study design and setting

An inductive exploratory design with a qualitative approach was chosen as the most fitting method for delving into the family members’ experiences of their everyday lives (Denzin and Lincoln, 2013). Data were generated through semi-structured interviews (McIntosh and Morse, 2015) with family members and analysed with a qualitative content analysis method (Elo and Kyngäs, 2008). To enhance transparency and rigour in the reporting of this qualitative study, the Consolidated Criteria for Reporting Qualitative Research (COREQ) checklist was used as a guiding framework (Tong et al., 2007).

### Participants and recruitment

The Swedish RLS patient organisation, with approximately 1,500 members, facilitated contact with individuals diagnosed with RLS. Initially, all organisation members were invited to participate in a questionnaire-based survey, with inclusion criteria requiring that participants must be over 18 years of age, diagnosed and treated for RLS, able to speak and understand Swedish and capable of providing written informed consent. Of the 788 members who consented and returned completed surveys, 472 (60%) indicated their willingness to have their family members contacted for a follow-up, qualitative, in-depth interview by ticking a box. Out of the 472 participants who agreed to be interviewed, we carefully selected 25 individuals with RLS to ensure a variety of connections to those with RLS (Creswell and Creswell, 2022). Background information gathered through questionnaires, such as gender, age, educational level, civil status, duration of RLS diagnosis (mean: 16.4 years), and RLS symptom severity (the majority of individuals with RLS had severe symptoms at night and during the day), guided this selection to maximise diversity. The individuals with RLS suggested and wrote the family members’ names and contact numbers on the information sheets. The chosen family members were then formally invited to participate based on the individuals’ characteristics in the current study. The goal was to introduce variation across key factors such as gender, age, education level, cohabitation, and the familial relationship to the individual with RLS, whether biologically or legally defined (e.g. partner, child). This selection process was guided by the interdisciplinary research team’s theoretical and clinical expertise regarding how RLS manifests across different contexts and levels of family connection (e.g. partner, child). The intention was to encompass a wide range of relevant family everyday experiences involving family members with diverse relationships to individuals with RLS.

### Interdisciplinary research team and reflexivity

The interdisciplinary research team consisted of ten members (four males, six females) with relevant academic and clinical credentials. They participated in study design, data collection, analysis, and interpretation. There were no prior relationships with participants, who were informed of the researchers’ roles, credentials and study aim before participation, following COREQ guidelines to improve rigour and transparency (Tong et al., 2007).

### Data collection

An informational letter detailing the study in general and the data collection process, that is the interview itself, was sent by mail to the 25 strategically selected family members (Table 1) using the contact details (e.g. name and phone number) provided by the individuals with RLS. The interdisciplinary research team made contact by telephone with the family members who agreed to participate. An interdisciplinary research team (i.e. including physicians and nurses) with extensive expertise in treating individuals with RLS and in qualitative analysis developed a semi-structured interview guide featuring open-ended and follow-up questions (Table 2). Two pilot interviews were conducted by two of the authors over the phone with family members and included in the analysis, as no changes to the interview content were needed after the pilot interviews (Creswell and Creswell, 2022). The family members were invited to share their experiences regarding their everyday lives and the support they received and gave. Clarifying questions were used throughout to ensure an in-depth exploration and to verify the interdisciplinary research team’s understanding of their responses. Four members of the interdisciplinary research team conducted the interviews by telephone between September 2022 and December 2023. The four interviewers and authors (E.O, M.J, M.G and L-L, J) brought extensive clinical experience as nurses, along with specialised skills in qualitative content analysis and in interviewing participants with chronic health conditions, and their family members. Fieldnotes were not taken during the interviews. Each interview, lasting 25–90 minutes, was audio-recorded and subsequently transcribed by a certified transcriptionist. The transcripts were not returned to the participants.

**Table 1.** Demographic of the family members (*N* = 25).

Variables	Value
Gender, female, *n* (%)	13 (52)
Age (years), mean (range)	65 (39–82)
Educational level, *n* (%)	
9 years or below	2 (8)
10–13 years	8 (32)
University	15 (60)
Relation to the patient with RLS, *n* (%)	
Partner (wife, husband)	23 (92)
Child	2 (8)
Diseases, *n* (%)	
Heart disease	4 (16)
Depression	3 (12)
Renal disease	2 (8)

**Table 2.** Questions included in the semi-structured interview guide with family members (*N* = 25).

Questions	Probing questions
Would you please share your experiences of what a typical day might look for you?	Could you please provide further explanation on the occurrence and experiences of symptoms through the day, tiredness and their effect on mood?
Would you please share your experiences of what a typical night might look for you?	Can you provide further explanation regarding the occurrence and experiences of nocturnal symptoms and how they affect your sleep?
Can you describe how you experience your life situation in relation to the patients RLS?	Can you explain that further relate to your work, family, social life, health, lifestyle, and living conditions?
Can you describe how you perceive what you can do to support the patient with RLS?	Can you provide further explanation about the specifics of the support you give?

### Data analysis

The interviews were analysed using qualitative content analysis according to Elo and Kyngäs’ model (2008). An inductive approach was applied to the interviews to identify categories, phrases, and keywords that represented the phenomena being described, specifically the experiences of family members of individuals living with RLS. The analysis process involved three steps: open coding, category creation, and abstraction analysis (Elo and Kyngäs, 2008), all guided by our research questions (Table 2). Open coding was carried out collaboratively by all authors, with each author re-reading the data, noting key concepts and writing descriptive labels in the margins. The data were then organised into sub-categories based on their relevance to family members’ everyday experiences. Four authors identified the sub-categories, which were then reviewed and refined by the remaining authors. Throughout this process, all authors engaged in discussions to resolve differences in interpretation, such as whether specific experiences should be classified under ‘Facing RLS-consequences’ or ‘Supporting the individuals with RLS’, and which participant’s quotes best illustrated each subcategory. Finally, all authors reviewed and refined the categories, reaching consensus on the overall categories and sub-categories (Table 3). Abstraction was then used to distil the findings into a main category. To ensure credibility (Lincoln and Guba, 1985), the interdisciplinary research team (all authors) discussed and reached a consensus on the final category system (Elo et al., 2014).

**Table 3.** Example of the data analysis process according to Elo and Kyngäs (2008), exploring and describing the everyday life experiences of family members of individuals with RLS (*N* = 25).

Research question	Quote	Code	Sub-category	Generic category	Main category
Would you please share your experiences of what a typical day might look for you?	*Spending time alone has become essential for me. It gives me the energy to focus on my own well-being or rest, yes, when I need to, and recharge before we are together again.* (Karin, partner, 52 years)	Prioritising personal time for well-being	Focusing on one’s own well-being activities	Adapting to the RLS-related consequences	RLS becomes integrated into the everyday lives of all family members
Would you please share your experiences of what a typical night might look for you?	*Of course, it was a bit tough for me as well when she couldn’t sleep and I was disturbed. She went to work tired, and I also felt exhausted, which made it hard since I had to get up and work too*. (Per, partner, 58 years)	Experiencing shared sleep disruption and tiredness	Addressing the individual’s RLS symptom during the nighttime	Facing RLS-related consequences	

### Ethical considerations

Ethical approval was obtained from the Swedish Ethical Review Authority (reference: 2022-01515-01). The family members received both written and verbal information about the study and its voluntary nature, and they were given time to ask questions and reflect before providing informed consent. To ensure confidentiality, the results are presented on a group level, and pseudonyms are used.

## Results

**Figure 1.**
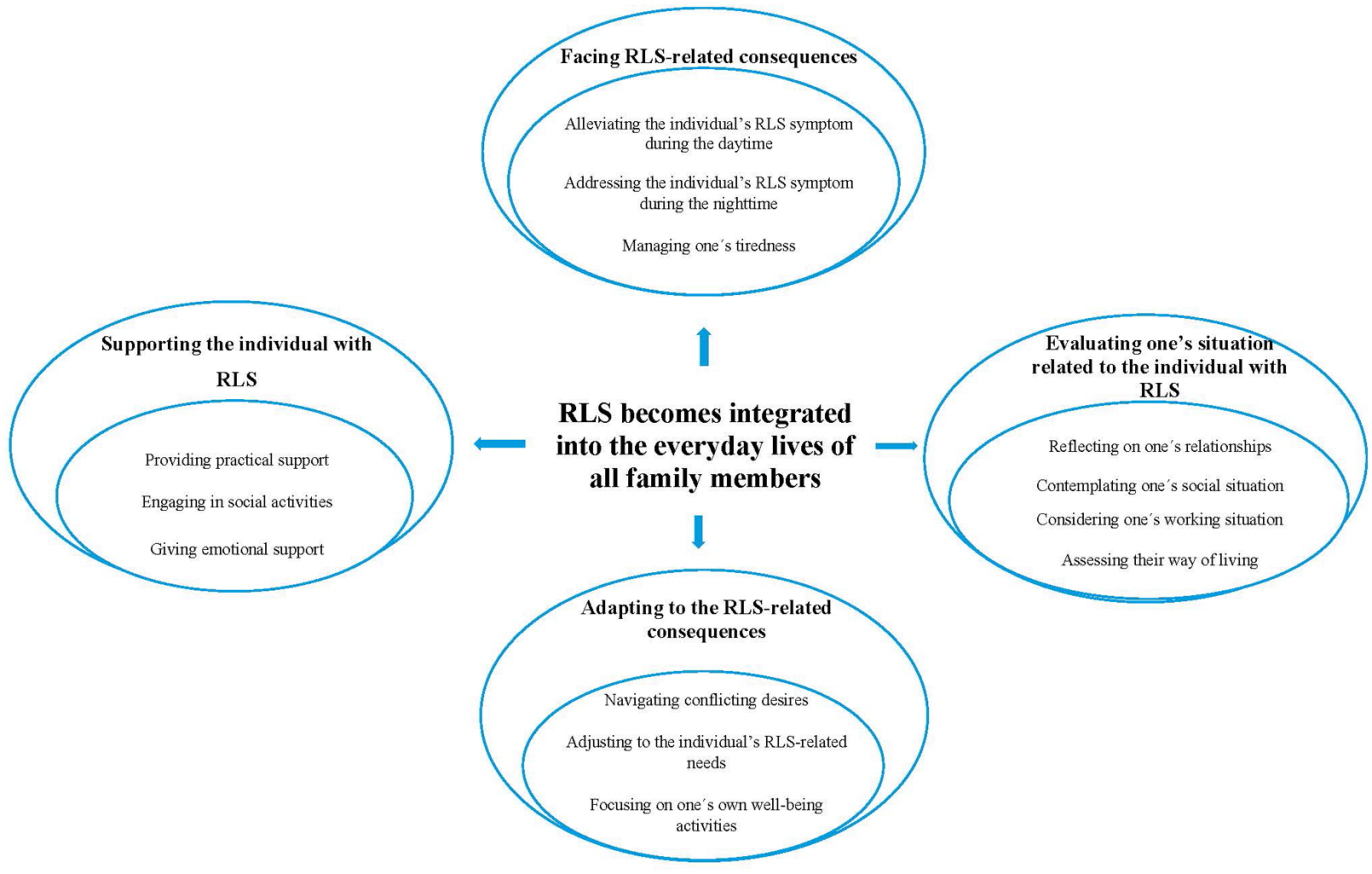
Overview of the categories and subcategories analysis structure.

The results included the main category ‘RLS becomes integrated into the everyday lives of all family members’ with 4 related generic categories and 13 sub-categories (Figure 1). All names presented next to the quotes are pseudonyms.

### RLS becomes integrated into the everyday lives of all family members

The significant impact of the individual’s RLS on family dynamics and routines led family members to face its challenges and disruptions. They tried to evaluate their own situations in relation to the condition, reflect on their own needs, adapt to the consequences of the individuals’ RLS in their everyday lives, and provide support within the family context.

### Facing RLS-related consequences

The family members accommodated and adapted to the individual’s RLS symptoms both during the day and at night, trying strategies to manage its impacts and cope with their own tiredness.

#### Alleviating the individual’s RLS symptoms during the daytime

The family members often found themselves adapting their daily routines to accommodate the effects of RLS. These adjustments included scheduling activities around times when the individual’s RLS symptoms were most intense and creating a home environment that allowed for frequent movement or stretching. They developed strategies to avoid planning long, uninterrupted activities, such as watching movies or taking extended car trips, knowing that frequent movement or walking would be necessary. This changed family routines, with daily activities, social events, and household chores organised around addressing these challenges.

Naturally, one tends to feel more tired and occasionally irritable before the day fully begins. It can be quite challenging. There’s also a certain sense of anxiety when trying to fall asleep, as one starts to wonder. `How much will my sleep be disturbed tonight?` It’s a thought that arises almost automatically (Birgitta-partner, 63 years)

#### Addressing the individual’s RLS symptom during the nighttime

The family members often had to adjust their routines and sleeping arrangements to cope with the impact of the individual’s RLS symptoms (e.g. leg movements, frequent awakenings) during the night. These symptoms disrupted their sleep, leading to frequent movement, awakenings, and the need to get out of bed in individuals with RLS. To manage these disruptions, family members opted for separate sleeping arrangements, such as using different bedrooms, to ensure they could rest without constant interruption. Additionally, they would wake up to offer support or assistance, such as helping with relaxation techniques or staying awake until the symptoms subsided. These nightly adjustments led to tiredness, frustration, and anxiety among partners, affecting their mood and the quality of the relationships.

Of course, it was a bit tough for me as well when she couldn’t sleep and I was disturbed. She went to work tired, and I also felt exhausted, which made it hard since I had to get up and work too (Per-partner, 58 years)

#### Managing one’s tiredness

The family members experienced significant tiredness caused by frequent sleep disruptions and the emotional demands of providing support. To address this tiredness, they adopted proactive self-care strategies, such as taking short naps during the day, safeguarding opportunities for uninterrupted rest, and seeking practical and emotional support from family and friends. This deliberate focus on self-care led them to re-evaluate their daily routines and make lifestyle adjustments, such as adding more flexibility to their schedules, changing leisure activities, and intentionally prioritising rest and recovery. Establishing regular routines further supported a balanced approach, helping them conserve energy, prevent burnout, and protect their well-being. By consistently attending to their self-care needs, family members felt better equipped to offer sustained and compassionate support to individuals with RLS while maintaining their own resilience.

During the night, his leg movements kept me awake, leaving me with only a few hours of sleep. I felt exhausted and had to take a nap in the afternoon after work, but the tiredness stayed with me throughout the rest of the day (Sofia-partner, 63 years)

### Evaluating one’s situation related to the individual with RLS

The family members reflected on, considered, and evaluated their situations regarding their relationships, social lives, and work conditions, all of which were affected by the individual’s RLS symptoms.

#### Reflecting on one’s relationships

The family members explained how the individuals’ RLS affected their relationships, both within their families and in their social circles. They noticed shifts in emotional connection (e.g. decreased quality time, loss of shared activities), communication, and daily interactions, as managing the challenges of the individuals’ RLS often introduced stress and strain into these relationships. There were family members (children), who noticed their parents’ RLS symptoms when they were children but did not gain any explanation of what their symptoms were about. Later, as adults, they reflected on what their parents had shared about their RLS symptoms, what they had experienced early on, and gained an understanding of experiences they had not recognised as children.

I never really knew about it; it’s only in the past few years that RLS has actually come up in conversation. She didn’t tell me when I was at home, like when I was 15 to 20. We just didn’t talk about it back then. She hasn’t complained about it either, but she also never said, ‘This is what’s going on, I have these issues’. So . . . yeah, what I really remember are those evenings (Flora-child, 39 years)

The family members also shared feelings of isolation, especially when others did not fully understand the difficulties they faced. Intimate relationships were also affected, with tiredness and disruptions creating challenges in maintaining physical closeness. This often led to feelings of distance or disconnection, resulting in frustration or a sense of loss. Reflecting on these experiences, family members sought ways to maintain healthy relationships and to find social and emotional support when needed.

But then there’s a consequence that I think is more serious, and it actually has to do with our sex life. Yes, I’ve thought a bit about this, you know, that . . . Yeah, but you know, that he’s tired and that he doesn’t have the energy (Stina-partner, 54 years)

#### Contemplating one’s social situation

The family members described how they often found themselves contemplating their social situation, reflecting on how the demands of managing the individuals’ RLS-related challenges affected their social lives. They felt restricted in participating in social activities or events, particularly when circumstances required their presence or frequent adjustments to plans (e.g. avoiding long events, cancelling plans at the last minute). This led to feelings of isolation, frustration and even guilt, as they tried to balance their own need for social interaction with these responsibilities. The family members recounted how their social lives had changed significantly over time. Before the challenges intensified, they had no trouble attending movies or social activities together. But as things became more difficult, those outings gradually stopped, and they began to miss these experiences. Watching films, whether at a cinema or at home, was challenging because frequent movement was often required, disrupting the experience. This lack of shared activities affected emotional closeness, as family members noticed their social lives had diminished due to tiredness and disruptions.

Well, what has changed a bit is the social aspect; it’s that you might not be able to meet as many people and that you don’t have as much of a social life (Mikael-partner, 75 years)

#### Considering one’s working situation

Family members often evaluated how the individual’s RLS symptoms affected their work. Disruptions from nighttime symptoms frequently led to tiredness, affecting their concentration, productivity, and overall energy levels at work. On some occasions, they found themselves needing to adjust their working hours, reduce their workload, or seek flexible arrangements to manage their personal well-being. Balancing professional obligations with the emotional and physical demands of support created additional stress, as they navigated the complexities of meeting both work and personal commitments.

My partner’s RLS at night affects my performance at work, and I also find it difficult when my partner is in pain. (Bertil-partner, 58 years)

#### Assessing their way of living

Family members described how the individual’s RLS symptoms led them to re-evaluate their way of living. They reflected on how the demands of the condition had reshaped their daily routines, limited their hobbies and reduced their personal time. This reflection often brought an awareness of how much their lives had changed and required ongoing effort to balance their own needs with those of the individual with RLS. They talked about adjusting their social lives to fit these changes, such as choosing quieter or more flexible activities that were easier to manage within their new limitations. These adjustments helped them maintain a sense of normalcy, even though they recognised their lifestyle had shifted significantly. Holidays or periods spent alone were described as a source of relief, offering a temporary respite from rigid schedules and the consequences associated with RLS. These periods accentuated the emotional contrast between everyday constraints and the experience of greater freedom resulting from fewer demands. Despite these efforts, the emotional impact of these lifestyle changes was clear. Family members expressed feelings of frustration, sadness, or resignation when reflecting on how these changes had affected their own lives.

Sometimes I feel angry; I can get really irritated and very upset with everyone because I wonder why it has to be this way. (Ingrid-partner, 68 years)

### Adapting to the RLS-related consequences

The family members adjusted to the demands imposed by the individual’s RLS, seeking to mitigate its consequences while also prioritising their own well-being.

#### Navigating conflicting desires

The family members faced ongoing tension as they tried to balance their own needs with the demands of their situation. They often felt torn between wanting to engage in different activities and the reality of staying home to manage the symptoms of the individual with RLS, which frequently worsened in the evenings. There were family members (children) who reflected on their childhood experiences of living with a parent with RLS, describing moments when they sought closeness and comfort, like sitting on the couch together, but realised their presence could irritate their parents. These early experiences from family members (children) illustrate that managing conflicting desires can begin in childhood. To cope, family members prioritised personal activities, such as exercise or hobbies, recognising that taking care of their own well-being helped them remain supportive. They also set boundaries around social commitments, choosing flexible gatherings or, when necessary, quieter evenings at home.

When I lived with my mother, sometimes we would sit on the couch watching a movie, and I’d put my legs on her lap. She would get irritated and start shaking her legs. As a teenager, I felt conflicted wanting to be close and comfortable, but also realising that my presence could make her uncomfortable. At the time, I didn’t fully understand how difficult it was for her (Tina-child, 42 years)

#### Adjusting to the individual’s RLS-related needs

Family members explained how the needs of the individual with RLS often shaped family routines and priorities. They frequently stayed home in the evenings to manage the individual’s RLS symptoms, which led to fewer social invitations and a decline in shared activities. Enjoyment of group activities decreased when the person struggled with symptoms, causing negative emotions that made caregiving and family life more difficult. Sleep disruptions caused by the individual’s RLS forced the cancellation of plans, reinforcing the pattern in which the individual’s needs always came first.

Before, we used to go to the movies and attend concerts and such things, but it feels like that’s over now (Alma-partner, 63 years)

#### Focusing on one’s own well-being activities

The family members described engaging in activities that primarily supported their personal well-being, such as reading, listening to music, meeting friends, or spending time alone or with others in nature. These activities were essential for recharging energy and maintaining emotional balance, allowing them to care for themselves without immediately addressing others’ needs. Periods of solitude, such as weekends or vacations, were particularly valued. During these times, family members structured their days around personal priorities, rested when needed, and engaged in enjoyable activities that fostered resilience.

Spending time alone has become essential for me. It gives me the energy to focus on my own well-being or rest, yes, when I need to, and recharge before we are together again. (Karin-partner, 52 years)

### Supporting the individual with RLS

The family members offered and provided practical, social, and emotional support to manage everyday life as ‘we’ in relation to the individual’s RLS.

#### Providing practical support

The family members described helping with practical tasks to facilitate everyday life, particularly when individuals with RLS felt symptom-related tiredness that was overwhelming. This practical support often included taking on extra household responsibilities, such as grocery shopping, cooking, or cleaning. Additionally, family members volunteered to drive or helped individuals with RLS drive to distract them from symptoms by focusing on something else. They also found that reminding the individual with RLS to take their medication helped prevent flare-ups resulting from missed doses. Other supportive actions included massaging the legs to alleviate symptoms or assisting with removing compression socks after prolonged wear.

Then, of course, I try to ease the everyday burden by often taking on tasks, like being the one who does the grocery shopping, cleaning, and handling things around the house (Hanna-partner, 53 years)

#### Engaging in social activities

The family members described how their social lives had been impacted by living with the individual with RLS. They often faced restrictions in participating in social events due to the need to modify plans or stay home, leading to feelings of isolation, frustration and guilt. Previously regular social outings, such as going to the movies or attending concerts, gradually stopped as RLS symptoms worsened, reducing opportunities for shared experiences and emotional closeness.

We actually started cold-water swimming last winter, and it’s been great for her legs. So it’s nice that we found something to do together as well (Ivar-partner, 70 years)

#### Giving emotional support

The family members described how learning more about RLS deepened their understanding of what individuals with RLS were experiencing, leading to emotional support. They recognised that while they offered limited practical help, the knowledge and awareness they gained allowed them to be more attuned to when the individuals’ RLS symptoms were present. This heightened sensitivity, combined with attentive and empathetic listening, fostered a more supportive, understanding relationship. The family members acknowledged that acceptance was key to making daily life smoother. They learned to adjust to the challenges and focus on making the best of the situation. They understood that although the symptoms were often severe and complex, it was impossible to fully comprehend their impact without experiencing them first-hand. With this understanding, their aim became to offer emotional support by making the individuals with RLS feel genuinely seen and heard. Adjusting to the situation without dramatising the symptoms was another way they eased the daily routine, fostering a sense of normalcy and acceptance.

I’ve also tried reading up on RLS to better understand what he’s going through, which has helped me to be more empathetic and patient (Anna-partner, 82 years)

## Discussion

This study employs a qualitative approach to explore and describe how family members experience everyday life alongside individuals with RLS, focusing on the impact of daytime and nighttime consequences on individual and family relationships and interactions. The results show that family members address the individuals’ RLS-related consequences by helping to alleviate symptoms while managing their own tiredness. Additionally, this study identified that RLS affected the family members’ social life, work and relationships, and the family members provide consistent social, emotional, and practical support to the individual with RLS.

Our study aligns with the FSN theory (Bell, 2009), demonstrating that family members living with individuals with RLS, which is a highly prevalent long-term illness (Broström et al., 2023), experience disruptions in their routines and relationships. These disruptions require adaptive strategies to manage everyday life. Specifically, given the unpredictable nature of RLS symptoms (Holzknecht et al., 2020), the family members in the present study described a need for frequent movement, walking and stretching, as responses to their partners increased RLS symptoms, which necessitated adjustments to the family members’ own plans. These alterations in family dynamics, such as scheduling activities around symptom flare-ups and modifying their environment, have been documented in various other long-term conditions (Marín-Maica et al., 2021; Pendoni et al., 2024), including Parkinson’s disease (Fekonja et al., 2024), heart disease (Andréasson et al., 2021) and mental illness (Fekadu et al., 2019).

In the present study, the family members developed adaptive strategies to manage RLS and its impact on sleep; these strategies, in turn, affected both their work and family life. It is a well-known fact that RLS adds another layer of stress, fatigue, and sleep disturbances to the lives of individuals suffering from the condition (Geng et al., 2022; Harrison et al., 2021; Odzakovic et al., 2024a; Odzakovic et al., 2025b). Consistent with these findings, a larger study (*n* = 676 spouses/partners of individuals with RLS) reported that 38% of family members experience impaired restful sleep, and 36% sleep in separate bedrooms to avoid disturbances (Ondo, 2018). In line with other studies, sleep quality assessed via the Pittsburgh Sleep Quality Index was poor in 85% of female spouses (*n* = 21) and 58.3% of male spouses (*n* = 14), with female spouses showing lower sleep efficiency, greater daytime sleepiness and higher rates of insomnia than male spouses (Say et al., 2021). However, there is still a gap in the literature on factors relevant to the development of sleep disorders in family members of individuals with RLS. The 3P Disease Model (Ellis et al., 2021; Wright et al., 2019) offers insight into how an individual’s vulnerability to stressors can influence the development of a chronic sleep disturbance. In our study, the family members described a combination of long-lasting stress related to the RLS diagnosis (i.e. a predisposing factor), as well as acute significant stress due to an increase in severe RLS symptoms during specific evenings and nights (i.e. a precipitating factor). This might have caused difficulties initiating and maintaining sleep for both the individuals with RLS, as well as the family members. If this continues over time, and treatment options fail to reduce symptoms (Guay et al., 2020, Winkelman et al., 2025), a chronic situation might develop for both parties (Wright et al., 2019). Therefore, the occurrence of sleep problems should be explored both in individuals with RLS and their family members, so that adequate information and support can be provided (Say et al., 2021). The components in the 3P model (i.e. the predisposing and precipitating factors and the perpetuating factor) could be focused on during such consultations. Future qualitative studies should investigate the relationships between RLS symptoms, fatigue, and sleep, particularly exploring how fatigue influences the impact on family members’ sleep quality.

The family members described how RLS affected their relationships, social lives, and work, leading to feelings of isolation and emotional strain. Despite these challenges, they prioritised self-care and flexibility in their everyday lives to maintain their well-being and relationships. These coping mechanisms included adjusting schedules and modifying expectations, which allowed them to nurture relationships and maintain a sense of normalcy. Previous research supports the idea that adaptive coping strategies such as re-prioritising social engagements and incorporating flexibility into daily routines are crucial for sustaining the mental health and well-being of family members (Daliri et al., 2024). Previous studies have emphasised the importance of acknowledging gender differences in enhancing the well-being and self-care of family members, as most caregivers are women (Swinkels et al., 2019). These findings align with previous research suggesting that women’s health may be more negatively affected by their roles as family members, with increased risk of isolation and depression (Swinkels et al., 2019). Although specific studies on how family members adapt to the effects of RLS are limited, existing research shows that relationships between individuals with RLS and their family members are vital (Ondo, 2018; Say et al., 2021). Even when family members are not the focus of studies, meaningful connections have been found between the support they offer and the psychological adjustment of both patients and their families (Fitzpatrick et al., 2024). It is essential to recognise the support needed by family members, both from other family members and through positive social connections, to reduce the risk of depressive symptoms (Walker & Reisig, 2024). In our study, family members were aware of how their physical health was affected, and feelings of isolation permeated their everyday lives, as the primary focus was often on the individuals with RLS rather than on their own physical health and needs such as intimacy and maintaining physical closeness. They acknowledged that this could create distance or a disconnect between partners, leading to frustration or a sense of loss. Therefore, discussions about both physical health and intimacy must be included during patients’ clinical appointment, family members should be asked about their own physical and sexual health and should receive adequate information and support.

The family members described the significant adjustments they had to make in response to the consequences of RLS, assuming roles that involved providing practical, social, and emotional support to the patient. Despite these challenges, they expressed gratitude for the opportunity to continue supporting each other and said they had developed various strategies to adapt to their new life circumstances. The experience of being valued and cared for by others is likely to enhance resilience to stressors and may even foster personal growth (Algoe and Zhaoyang, 2016). In our study, the family members emphasised the importance of engaging in activities that supported their well-being, such as spending time with friends, reading, and listening to music. They also highlighted the critical need for personal time to recharge and to provide an opportunity to focus on their own needs and enjoy activities that allowed them to disconnect from caregiving responsibilities. This balance between caring for the individual and caring for themselves was essential for maintaining their mental and emotional health. To maintain stability within the family, coping strategies are necessary for managing life alongside a long-term illness (Newby, 1996). This perspective aligns with the Resiliency Model of Family Stress, Adjustment and Adaptation by McCubbin and McCubbin (1983), which suggests that families draw on psychosocial resources (such as family attributes and relationships), tangible resources (like family possessions), and coping behaviours to navigate stressful events. This study highlights how these coping strategies play a vital role in helping family members adapt to the ongoing challenges posed by RLS symptoms and their impact on everyday life. Furthermore, our findings provide valuable insights into the everyday lives of family members, offering new implications for clinical practice.

A more nuanced understanding of family members’ experiences is vital, as nurses sometimes overlook the role of family members during patient appointments. Such awareness is particularly important from a family nursing perspective (Bell, 2009; Wright et al., 1985), where nurses should recognise the challenges family members face and involve them in the care process. Family involvement in the decision-making process concerning RLS care is essential, especially since individuals usually do not manage their condition alone. It is important to pay attention to both the patient and their family simultaneously. The insights provided by the patient and their family members, combined with the doctors’ interpretation, are crucial for accurate diagnosis and treatment decisions (Fulda et al., 2021). Involving family members in the patient’s care can create both opportunities and challenges that influence their participation in decision-making, while also highlighting the significant impact of the condition beyond the patient, since family members often experience disrupted sleep, emotional strain and increased burdens (Ondo, 2018; Say et al., 2021). Despite the availability of educational resources from organisations such as the Restless Legs Syndrome Foundation, structured support for family members remains limited. Routine clinical appointments primarily focus on patient symptoms and treatment (Winkelmann et al., 2025), rarely addressing the physical or mental well-being of family members. This highlights the importance of care approaches that actively recognise and support families. Incorporating FSN principles (Bell, 2009) into RLS care can improve communication and ensure consistent attention to family needs and shared decision-making (SDM). Enhancing their understanding of the process could facilitate SDM in practice (Elwyn, 2021). SDM is a vital component of person-centred care (PCC) and is key to achieving optimal outcomes (Elwyn, 2021), particularly in the context of RLS (Björk et al., 2024), where family members play an active role in decisions regarding the patient’s care. However, involving family members presents challenges, such as time constraints, confidentiality concerns and role ambiguity in expressing preferences (Habermann et al., 2020; Steffensen and Berry, 2025). Applying FSN principles can support SDM, but practical barriers must be acknowledged. Educating families and healthcare professionals, along with structural supports like sufficient consultation time and training (Steffensen and Berry, 2025), is crucial to making SDM feasible and sustainable in routine RLS care. Therefore, it is important to educate both families and healthcare professionals on how to improve partnership and communication (Wright and Leahey, 2009) throughout the RLS care process (Harrison et al., 2021).

### Strengths and Limitations

There are several limitations to consider. Firstly, the family members were selected from a patient organisation, which may have impacted the findings. The family members, along with the individuals, may have been more engaged in RLS care compared to those not involved in a patient organisation. However, a particular strength is that our data are based on 25 family members, comprising a relevant and plausible sample size for a strategic selection in a qualitative study. The sample also included variations in age, gender, and education, which reflected the demographics of family members.

Although the study included children of individuals with RLS, a group rarely examined in previous research, only two children participated. This small sample limits the diversity of perspectives and the transferability of findings, especially regarding children’s experiences. Nonetheless, their participation provided valuable, previously undocumented, insights that reflected both their childhood and adult viewpoints. Including their voices highlights the vital role children play within the family system and helps build a more comprehensive understanding of how RLS impacts family life. Their inclusion can also be seen as a strength of the study, as it captures overlooked perspectives that add depth and nuance to the results. This increases the credibility and transferability of the results. Secondly, the use of telephone interviews is often seen as a less attractive alternative to face-to-face interviews due to the absence of visual cues, which can result in the loss of contextual and nonverbal data that might affect the probing and interpretation of responses (Novick, 2008). However, it is very possible that telephone interviews have helped family members feel more relaxed and comfortable. Finally, a limitation could be the potential influence of the four interviewers who conducted the interviews. However, the risk of discrepancies in our data collection was mitigated through extensive discussions within the interdisciplinary research team both before and after data collection as well during data analysis. This allowed for diverse perspectives on the issue under study, thereby enhancing dependability and confirmability.

## Conclusion

The findings shed light on the challenging everyday life faced by family members who evaluate, adjust to, and support individuals with RLS. They underscore the importance of utilising appropriate coping strategies, such as re-prioritising activities and maintaining flexibility in everyday routines, to manage the impact of RLS on family life. Moreover, the results highlight the need for healthcare professionals to consider the well-being of family members, particularly regarding sleep disturbances and emotional health, alongside the patient’s needs. This study also emphasises the significance of family involvement in RLS care and SDM, reinforcing the value of a family nursing approach. Future studies should explore and describe mental health and social support to gain a comprehensive understanding of family members’ experiences, and it is also crucial to examine the dyadic perspective of couples living with RLS and how it affects their everyday lives.

**Key points for policy, practice and research**

- Healthcare professionals should actively involve family members in clinical consultations and SDM to ensure both patients’ and families’ needs are recognised.
- Healthcare policies and practices could provide family members with resources and education to develop suitable coping strategies that sustain family functioning, decrease isolation, and focus on their own well-being.
- A family nursing approach should be integrated into clinical guidelines and community services to acknowledge gendered caregiving roles, enhance resilience, and strengthen supportive networks.
- Future research should focus on mapping the social support available to family members and the dyadic perspective of couples living with RLS, as well as its impact on their daily lives. This involves integrating specific theories, such as FSN theory and Social Support theory, and using both qualitative and quantitative research methods, since there is a lack of detailed knowledge in this area.
